# High rates of SARS-CoV-2 reinfection in residents of long term care facilities despite robust spike-specific immunity following serial vaccination

**DOI:** 10.1371/journal.pone.0354079

**Published:** 2026-08-14

**Authors:** Gokhan Tut, Tara Lancaster, Panagiota Sylla, David Bone, Christopher Bentley, Azar Jadir, Rachel Bruton, Katie Spencer, Soumyajit Mallick, Ahmed Elzaidi, Alexander Dowell, Mary Wu, Ruth Harvey, Edward Carr, Rupert Beale, Maria Krutikov, Oliver Stirrup, Borscha Azmi, Andrew Hayward, Andrew Copas, Laura Shallcross, Paul Moss, Crick COVID Immunity Pipeline

**Affiliations:** 1 Department of Immunology and Immunotherapy, University of Birmingham, Birmingham, United Kingdom; 2 Covid Surveillance Unit, The Francis Crick Institute, London, United Kingdom; 3 Worldwide Influenza Centre, The Francis Crick Institute London, London, United Kingdom; 4 The Francis Crick Institute London, London, United Kingdom; 5 Genotype-to-Phenotype UK National Virology Consortium (G2P-UK), London, United Kingdom; 6 UCL Department of Renal Medicine, Royal Free Hospital, London, United Kingdom; 7 UCL Institute of Health Informatics, London, United Kingdom; 8 UCL Institute for Global Health, London, United Kingdom; 9 United Kingdom Health Security Agency, London, United Kingdom; Queensland Health, BANGLADESH

## Abstract

Older adult residents of long-term care facilities (LTCFs) suffered high rates of mortality during the initial stages of the COVID-19 pandemic but their clinical risk has decreased markedly following vaccination. Here we determined humoral and cellular immunity following delivery of a 5th vaccine dose, an mRNA spike B1:BA.1 bivalent vaccine, to care home residents. The delivery of a 5th vaccine elicited a plateau of spike-specific immunity that remained broadly stable over 100 days in almost all people. Despite this, 15% of residents had a primary infection and 30% became reinfected during 6-months of follow up. These findings reveal that serial vaccine delivery can establish robust systemic spike-specific immune responses in frail older people but that this does not reliably prevent SARS-CoV-2 reinfection. As such, additional approaches should be considered to reduce reinfection risk in this vulnerable population group.

## Introduction

Age and frailty are important determinants of the clinical severity of COVID-19. Residents living in long-term care facilities (LTCFs) were at particular risk, with high rates of mortality observed prior to the introduction of SARS-CoV-2 vaccines. Vaccination provided strong clinical protection in this group although immune responses following primary series vaccination were suboptimal and required enhancement through booster doses [1]. Furthermore, although vaccination attenuated the clinical burden of infection, the infection-related mortality rate remained elevated in large national studies in 2023 [2,3] although more reassuring outcomes have been observed in other settings. In particular, the increased infection prevalence in Italian nursing homes following evolution of the Omicron variant was not associated with increased mortality rate [4] and a 4^th^ vaccine dose provided protection against mortality in Sweden [5].

The emergence of the Omicron SARS-CoV-2 variant led to the deployment of bivalent or Omicron-specific vaccines which have demonstrated encouraging Omicron-specific immunogenicity in a range of studies, including the LTCF setting [6]. Nevertheless, detailed assessments of immunogenicity in such vulnerable populations remain limited and have not been related to risk of subsequent infection or reinfection over the study period [7]. This information is of value both to assess the justification for continuation of the vaccination programme as well as assessing if such a programme alone is sufficient to minimise reinfection rates.

We determined adaptive immune responses against spike protein following bivalent vaccination in the LTCF setting. Delivery of 5 vaccines was seen to provide a stable immune platform of antibody and cellular responses against several Omicron variants, largely independent of prior infection status. Despite this, infection rates remain high and indicate a potential need for additional protective approaches.

## Materials and methods

### Sample collection

The VIVALDI study (ISRCTN14447421) is a prospective cohort study which was set up to investigate SARS-CoV-2 transmission, infection outcomes and immunity in residents and staff in LTCFs in England that provide residential and/or nursing care for adults aged 65 years and over (https://wellcomeopenresearch.org/articles/5-232/v2).

LTCFs were identified by the Senior Management Team, or by the National Institute for Health and Care Research (NIHR) Clinical Research Network. Pseudonymised clinical (vaccination status, PCR/LFD test results, hospitalisation, death) and demographic (age, sex, staff member versus resident) data were retrieved for staff and residents from participating LTCFs through national surveillance systems. All participants provided written informed consent for blood sample collection or if residents lacked the capacity to consent, a personal or nominated consultee was identified to act on their behalf.

Blood sampling was carried out from 27th July 2022 until 19th of January 2023, aligning with the administration of the bivalent vaccine. Ethical approval for this study was obtained from the South Central – Hampshire B Research Ethics Committee, REC Ref: 20/SC/0238.

### Data linkage

Roche anti-nucleocapsid antibody test results were submitted to the COVID-19 datastore (https://data.england.nhs.uk/covid-19/), pseudonymised and linked to routinely held data on age, sex, LTCF, role (staff or resident), and results of PCR or lateral flow device (LFD) SARS-CoV-2 testing performed through the national SARS-CoV-2 testing programme (REF). Using the common pseudo-identifier based on the individuals’ NHS number, linkage was undertaken to vaccination status (date and vaccine type) derived from the National Immunisations Management System (NIMS) and dates and diagnostic codes for hospitalisations recorded in the Hospital Episode Statistics (HES) dataset as well as for any deaths from the Office for National Statistics (ONS) dataset. Individual-level records were further linked to each LTCF using the unique Care Quality Commission location ID (CQC-ID) recorded on PCR/LFD tests, allocated by the Care Quality Commission who regulate all providers of health and social care in the UK. The legal basis for accessing data without consent was initially under the Control of Patient Information Regulations 2002 (notice in place between March 2020 and June 2022). The study obtained section 251 support from the Health Research Authority’s Confidentiality Advisory Group (CAG) (ref 21/CAG/0156) in March 2022.

### Inclusion criteria

Staff and residents were eligible for inclusion if samples could be linked to a pseudo-identifier enabling data linkage. We included samples from participants that had received a primary vaccine course of two doses, with or without a third, fourth and fifth dose. Fourth and fifth dose administration coincided with the introduction of the bivalent vaccine. Samples obtained within 6 days of booster vaccine were excluded to ensure peak immune responses were reached. Due to limited PCR testing up to July 2020 and after March 2022 it was not possible to determine when individuals had been infected with SARS-CoV-2 based on PCR/LFD alone. Past infection with SARS-CoV-2 was defined based on results of MSD nucleocapsid antibody test and Abbotts test using thresholds and methods outlined below. No statistical methods were used to pre-determine sample size but participant numbers are similar to those reported in previous publications [8–11].

298 participants (141 residents and 157 staff) received a third vaccine. Median time between administration of the third vaccine dose was 333 days for residents (IQR 285−401) and 363 days for staff (IQR 328−408). Out of the 298 recipients of three COVID-19 vaccine doses, 5 residents and 45 staff did not receive further doses of COVID-19 vaccine.

A total of 195 participants (135 residents and 60 staff) received a 4^th^ dose of COVID-19 vaccine. The median time between the 4th vaccine and sample collection was 63 days for staff (IQR 41–99) and 133 days for residents (IQR 106–205). 67 participants (22%) received a 5th dose of COVID-19 vaccine consisting of 64 residents and 3 staff members, The median time to sample collection was 30 days for staff (IQR 26–67) and 65 days for residents (IQR 31–104).

Out of 141 LTCF residents, 30 provided matched samples, once after administration of a 4^th^ dose of COVID-19 vaccine and once after receiving a 5^th^ dose of COVID-19 vaccine.

### Sample preparation

Anticoagulated blood samples were processed within 24 hours and PBMC isolated using SepMate (Stemcell) density centrifugation tube and rested overnight in R10 (RPMI + 10% FBS + Pen/Strep) media at 37°C in 5% CO2. Plasma and serum samples were assessed antibody binding and viral neutralisation.

### Serological analysis of SARS-CoV-2-specific immune response

Quantitative IgG antibody titres were measured against Spike (S) and Nucleocapsid (N) proteins using the MSD V-PLEX COVID-19 IgG Kit (SARS-CoV-2 Panel 2, 25 and 27) (Lot number K0081795) in line with prior reports [1]. Past infection with SARS-CoV-2 was defined by a Nucleocapsid (N) IgG titre of 1200 AU/ml. Data was generated by Methodical Mind software and analysed with MSD Discovery Workbench (v4.0) software. Presented data were adjusted for any sample dilutions.

### Quantification of SARS-CoV-2-specific cellular responses

Pepmixes pool containing 15-mer peptides overlapping by 10aa from either SARS-CoV-2 Wuhan or Omicron BA.1 or BA4/5 Spike S1 were purchased from JPT Peptide Technologies (Germany). ELISpot analysis was used to assess T cell responses with the IFN/IL-2 FluoroSpot Plus Kit (Mabtech, Sweden). 2-3x10^5^ PBMC were used in assays as previously described [1]. Cytokine concentrations within ELISpot supernatants were assayed using a LEGENDplex™ COVID-19 Cytokine Storm Panel 1 (BioLegend, Lot Number B332349) and analysed using the LEGENDplex™ Data Analysis Software Suite (BioLegend).

### High-throughput live virus microneutralisation assay

The B.1.617.2 (“Delta”) isolate was MS066352H (GISAID accession number EPI_ISL_1731019), and was kindly provided by Prof. Wendy Barclay, Imperial College London, London, UK through the Genotype-to-Phenotype National Virology Consortium (G2P-UK). The BA.1 (“Omicron”) isolate was M21021166, and was kindly provided by Prof. Gavin Screaton, University of Oxford, Oxford, UK through the Genotype-to-Phenotype National Virology Consortium (G2P-UK).

Viral isolates were propagated in Vero V1 cells. Briefly, 50% confluent monolayers of Vero V1 cells were infected with the given SARS CoV-2 strains at an MOI of approx. 0.001. Cells were washed once with DMEM (Sigma; D6429), then 5 ml virus inoculum made up in DMEM was added to each T175 flask and incubated at room temperature for 30 minutes. DMEM + 1% FCS (Biosera; FB-1001/500) was added to each flask. Cells were incubated at 37° C, 5% CO2 for 4 days until extensive cytopathogenic effect was observed. Supernatant was harvested and clarified by centrifugation at 2000 rpm for 10 minutes in a benchtop centrifuge. Supernatant was aliquoted and frozen at −80°C. Full protocol for variant virus culture and microneutralization assay available from Wu *et al.* 2022 [12].

High-throughput live virus microneutralisation assay was run as previously described [13]. Specifically, VERO E6 cells (Institut Pasteur) at 90−100% confluency in 384-well plates (Greiner) were infected with SARS-CoV-2 variants at an MOI of <1 in the presence of patient serum samples in serial dilutions. Cells were fixed with 4% final formaldehyde, blocked and permeabilised with 3% BSA + 0.2% TritonX-100 in PBS (v/v), and infected cells stained using a Biotin-CR3009 antibody (produced in-house), which specifically detects SARS-CoV-2 N-protein, and Streptavidin-Alexa488 (Invitrogen). Cellular DNA detected using DAPI. An Opera Phenix (Perkin Elmer) automated microscope was used to image whole wells at 5x and the fluorescent areas calculated using the Phenix-associated software Harmony (Perkin Elmer). The sample IC50 against a variant was estimated by fitting a 4-parameter dose response curve using SciPy and reported as the fold-dilution of serum samples required to inhibit 50% of detected infection, with additional annotation if the result lies outside the quantitative range (complete inhibition, no inhibition, weak inhibition).

### Statistical analysis

All data were checked for normality using the Kolmogorov-Smirnov test. For comparative analysis of 2 groups a Mann Whitney test was applied. For paired data, 2-tailed paired t-tests (parametric) or Wilcoxon matched-pairs signed rank tests (non-parametric) were applied. For comparative analysis with 3 or more groups a Kruskal-Wallis test was used, and for multiple comparisons uncorrected Dunn’s test was used for non-parametric data. Spearman’s rank correlation coefficients were calculated and tested for correlations. P values <0.05 were considered to be statistically significant.

**Fig 1:**
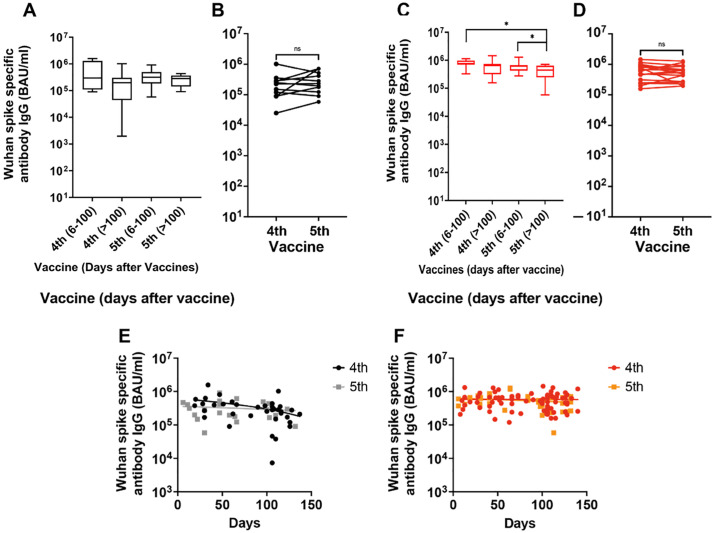
Spike-specific antibody titres plateau after 5 vaccines and remain stable over 100 days in LTCF residents. (A) Spike-specific antibody titre following the fourth and fifth SARS-CoV-2 vaccine doses in non-prior-infected long-term care facility (LTCF) residents. Antibody responses were analysed using a linear mixed-effects model with participants included as a random effect. Tukey-adjusted pairwise comparisons identified no significant differences between time points (Early_4 vs Late_4, p = 0.9931; Early_4 vs Early_5, p = 0.9308; Early_4 vs Late_5, p = 0.9999; Late_4 vs Early_5, p = 0.9541; Late_4 vs Late_5, p = 0.9229; Early_5 vs Late_5, p = 0.8496). Data are shown as box-and-whiskers plots, where the centre line represents the median, the box represents the interquartile range (IQR), and whiskers indicate the minimum and maximum observed values. A total of 40 observations from 33 individuals were included in the analysis. (B) Spike-specific antibody titre after 4th and 5th vaccine in matched non-prior infected LTCF residents. Paired T test p = 0.52 n = 11. (C) Spike-specific antibody titre following the fourth and fifth SARS-CoV-2 vaccine doses in prior-infected long-term care facility (LTCF) residents. Antibody responses were analysed using a linear mixed-effects model with participants included as a random effect. Tukey-adjusted pairwise comparisons identified significant differences between Early_4 and Late_5 (p = 0.0242) and between Early_5 and Late_5 (p = 0.0347). However, no significance was observed in the remaining comparisons (Early_4 vs Late_4, p = 0.1838, Early_4 and Early_5, p = 0.6478, Late_4 and Early_5, p = 0.3438, Late_4 and Late_5, p = 0.3086). Data are shown as box-and-whiskers plots, where the centre line represents the median, the box represents the interquartile range (IQR), and whiskers indicate the minimum and maximum observed values. A total of 94 observations from 77 individuals were included in the analysis. (D) Spike-specific antibody titre after 4th and 5th vaccine in matched prior infected LTCF residents. Paired T test p = 0.52, n = 19. (E) Spike-specific antibody titre in relation to days after 4th or 5th vaccine in LTCF residents with no prior infection. Black indicates 4th vaccine (Two-tailed Spearman’s correlation r = −0.404, p = 0.014, n = 36). Grey indicates 5th vaccine (Two-tailed Spearman’s correlation r = −0.139, p = 0.535, n = 22) All fitted lines are linear regressions. (F) Spike-specific antibody titre in relation to days after 4th or 5th vaccine in LTCF residents with prior infection. Red indicates 4th vaccine (Two-tailed Spearman’s correlation r = 0.030, p = 0.779, n = 86). Orange indicates 5th vaccine (Two-tailed Spearman’s correlation r = −0.159, p = 0.294, n = 45). All fitted lines are linear regressions.

In Fig 1, matched samples were collected at significantly different intervals following the fourth and fifth vaccine doses. To assess whether equivalent post-vaccination sampling windows were compared between the fourth and fifth vaccine doses, the number of days between vaccination and sample collection was calculated for each sample. For samples assigned to the Early_4 and Late_4 cohorts, days post-vaccination were calculated relative to the fourth vaccine dose. For samples assigned to the Ealy_5 and Late_5 cohorts, days post-vaccination were calculated relative to the fifth vaccine dose. Sample intervals were compared between the Early_4 and Early_5 cohorts and between the Late_4 and Late_5 cohorts using the paired Wilcoxon signed-rank Test.

For analyses that included paired and unpaired data, with more than 3 groups, a linear mixed-effects model was used with participants as a random effect, and then Tukey-adjusted pairwise comparisons were used to correct for multiple testing.

Samples assigned to the fourth-dose cohort were collected at significantly different post-vaccination intervals than samples assigned to the fifth-dose cohort (paired Wilcoxon signed-rank test, V = 431.5, p < 0.001).

## Results

### Spike-specific antibody responses plateau after 5 vaccines in LTCF residents

309 participants from long-term care facilities (LTCFs) were recruited of which 168 were staff and 141 residents (Table 1). Blood samples were collected after the 3rd, 4th or 5th vaccine. Responses after 5^th^ vaccine in staff members were not assessed as only 3 received the vaccine due to their lower age and clinical risk.

**Table 1:** Donor demographics.

	Staff	Resident	Total (%)	Total with prior SARS-CoV-2 infection (%)
**Total vaccine participants**	168	141	309 (100)	219 (70)
**Total 5th vaccine participants**	3	64	67 (22)	45 (67)
**Age >=80**	2	118	120 (39)	81 (68)
**Age 65–79**	18	21	39 (13)	28 (72)
**Age <= 64**	148	2	150 (48)	110 (73)
**Median age in years (IQR)**	51 (40-59)	89 (84-93)		
**Female**	141	96	237 (77)	168 (71)
**Male**	27	45	72 (23)	51 (71)
**Baseline mRNA vaccine recipients**	131	68	199 (64)	137 (69)
**Baseline ChAdOx1 recipients**	37	73	110 (36)	82 (75)
**Median days between 4th vaccine and sample (IQR)**	63 (41-99)	133 (106-205)		
**Median days between 5th vaccine and sample (IQR)**	30 (26-67)	65 (31-104)		
**Total LTCFs**	25			
**Median participants/LTCF (SD)**	9 (9.5)			

**(+** indicates SARS-CoV-2 prior infection).

Vaccine dose and prior infection status were then related to SARS-CoV-2 spike-specific antibody response and titres were seen to plateau after the 4th or 5th vaccine, irrespective of prior infection status. Hybrid immunity limited antibody waning after the 4th vaccine but levels stabilised in LTCF residents following 5 vaccines (Fig 1). Of note, the median time between the 4th dose and sampling for residents was 133 days, compared to only 65 days after 5th dose, and so further longer term follow up will be of interest.

As such, antibody levels plateau after 5 vaccines and remain broadly stable over the next 100 days irrespective of prior infection status.

### Bivalent vaccine increases antibody titres against Omicron variants in LTCF residents and enhances neutralisation in infection-naive donors

**Fig 2:**
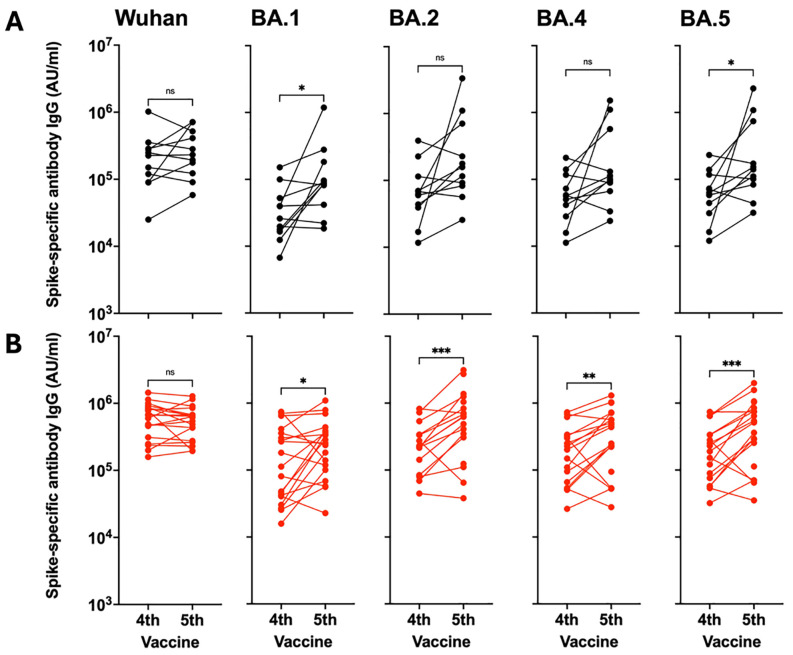
Omicron -specific antibody responses are increased following bivalent vaccination in LTCF residents. A. Ancestral, BA.1, BA.2, BA.4 and BA.5 spike-specific antibody responses after 4th and 5th dose vaccine in non-prior infected LTCF residents. Lines indicate paired samples. Wilcoxon matched-pairs signed rank test; Wuhan p = 0.58, BA.1 p = 0.02, BA.2 p = 0.06, BA.4 p = 0.12, BA.5 p = 0.04, n = 11. B. Ancestral, BA.1, BA.2, BA.4 and BA.5 spike-specific antibody responses after the 4th and 5th dose vaccine in prior infected LTCF residents. Lines indicate paired samples. Wilcoxon matched pairs signed rank test; Wuhan p = 0.3, BA.1 p = 0.03, BA.2 p = 0.0003, BA.4 p = 0.002, BA.5 p = 0.0004, n = 19.

We next determined spike-specific antibody responses against the SARS-CoV-2 Omicron variants BA.1, BA.2, BA.4 and BA.5 in matched samples from elderly LTCF residents following the 4th and 5th (B1 and BA.1 bivalent) vaccine dose. Titres against BA.1 and BA.5 were increased in both groups following bivalent vaccination (Fig 2A).

**Fig 3:**
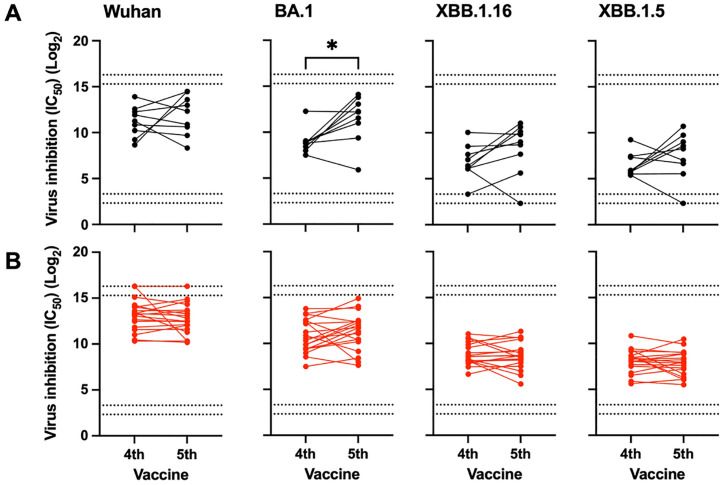
Bivalent vaccine increases BA.1 specific virus neutralisation in LTCF residents. A. Matched antibody neutralisation of ancestral Wuhan, BA.1, XBB.1.16 and XBB1.5 variants from LTCF residents with no prior infection shown after 4th and 5th vaccines. The bottom and top dotted-lined regions indicate weak and complete inhibition, respectively. Wilcoxon matched-pairs signed-rank test; Wuhan p = 0.49, BA.1 = p.027, X.BB.1.1 p = 0.97, XBB.1.5 p = 0.25. B. Matched antibody neutralisation of ancestral Wuhan, BA.1, XBB.1.16 and XBB1.5 variants from LTCF residents with prior infection shown after 4th and 5th vaccines. The bottom and top dotted-lined regions indicate weak and complete inhibition, respectively. Wilcoxon matched-pairs signed-rank test: Wuhan p = 0.44, BA.1 p = 0.13, XBB.1.6 p = 0.622, XBB.1.5 p = 0.51.

Relative viral neutralisation capacity of post-vaccine sera following 4th and 5th vaccines was also assessed [13]. 5th vaccine enhanced BA.1-specific neutralisation in infection-naïve donors (Fig 3A). This trend was also observed in the non-linked data set (S1 Fig). Some increase in neutralisation of the XBB.1.16 and XBB.1.5 variants was seen after both vaccines, most particularly in infection-naive donors with values suggestive of effective neutralisation capacity *in vivo*.

As such, bivalent vaccine enhances omicron variant-specific antibody titres irrespective of infection status and further enhances neutralisation capacity in donors who remain infection-naive.

### T cell responses are robust following 5th vaccine with reduced recognition of BA.5

**Fig 4:**
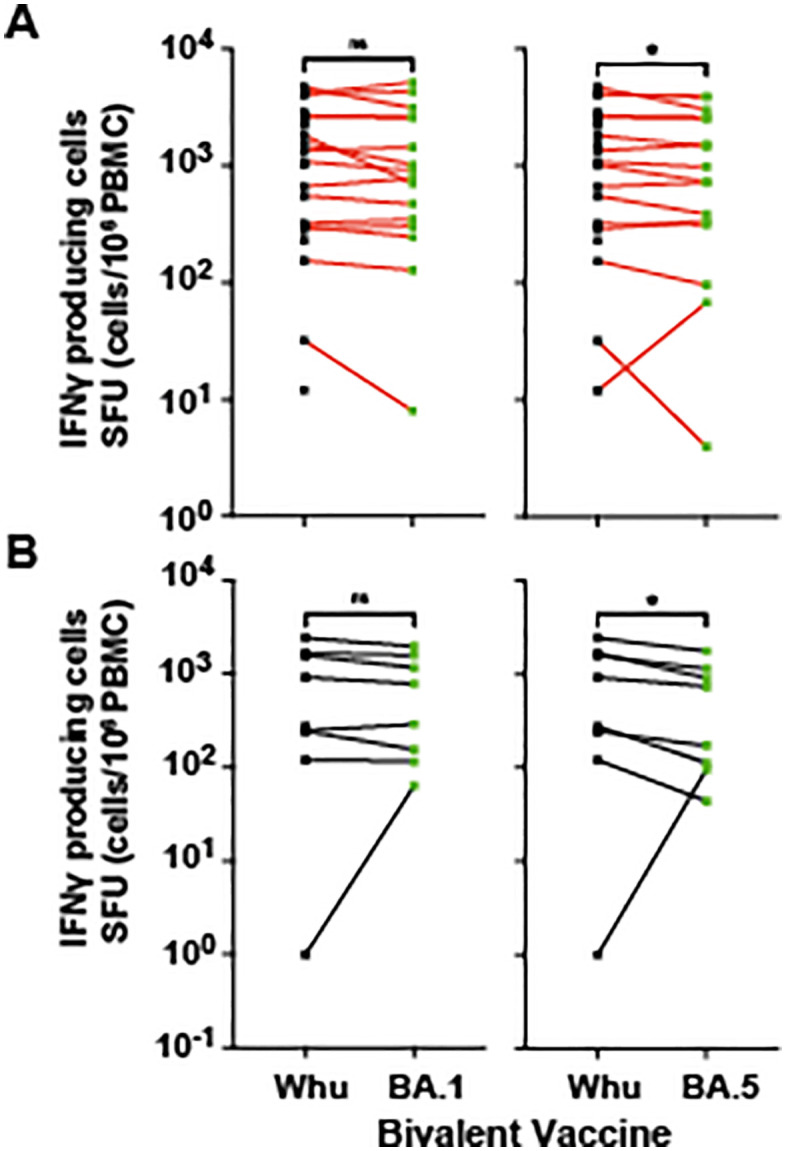
Robust spike-specific cellular responses in LTCF residents. A. Wuhan, BA.1 or BA.5 spike S1-specific IFN-γ cellular response after bivalent vaccine in prior infected LTCF residents. Black dots represent Wuhan response and green dots represent BA.1 or BA.5 responses. Wilcoxon matched-pairs signed rank test (Wuhan to BA.1 p = 0.33, Wuhan to BA.5 p = 0.02 (n = 16)). B. Wuhan, BA.1 or BA.4/5 spike S1-specific IFN-γ cellular response after bivalent vaccine in non-prior infected LTCF residents. Black represents Wuhan response and Green represents BA.1 or BA.4/5 responses. Wilcoxon matched-pairs signed rank test (Wuhan to BA.1 p = 0.08, Wuhan to BA.5 p = 0.04 (n = 8)).

T cell responses against spike protein from ancestral virus and Omicron variants BA.1 and BA.5 were also assessed following the bivalent vaccine (Fig 4A/B). Recognition of ancestral and BA.1 peptides was equivalent whilst those against BA.5 were slightly reduced, irrespective of prior infection status, although sample size was modest in the non-infected group.

### Serial vaccination incrementally supports adaptive immunity protection with additional vaccine doses required for infection-naive donors

**Fig 5:**
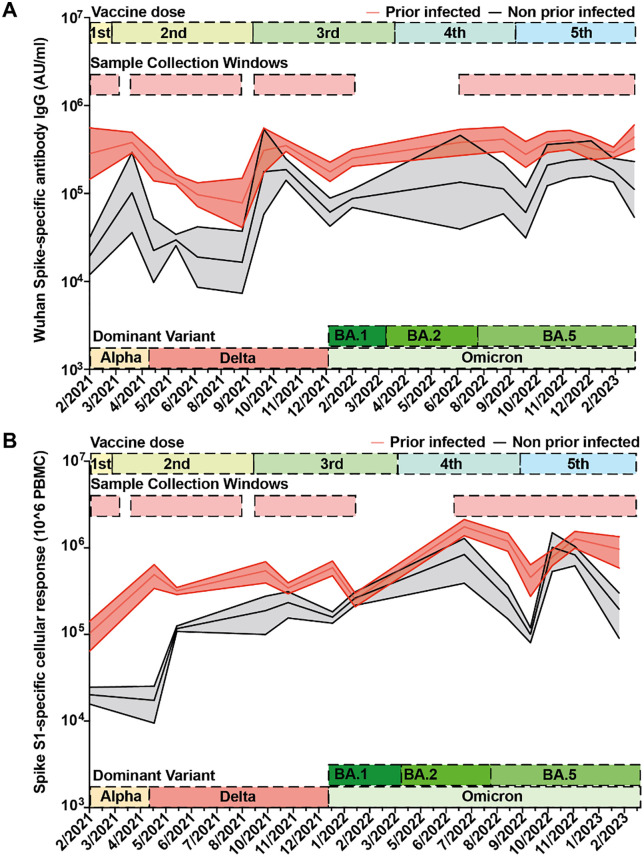
Humoral and cellular responses following SARS-CoV-2 vaccination during VIVALDI study. (A) Spike-specific antibody or (B) Spike S1 domain cellular responses from all participants in the VIVALDI study during the collection period. Red indicates prior infected donors and black indicates those that remained infection-free. Standard error of the mean (SEM) is shown. The time periods of delivery of the 5 vaccine doses are shown together with times of sample collection. The most dominant SARS-CoV-2 variant that was circulating during sample collection is also represented.

Antibody and cellular response had been assessed incrementally in LTCF residents and staff within the VIVALDI study since initial vaccination. These prospective findings showed that repeat vaccination had served to deliver robust spike-specific immunity to almost all donors (Fig 5).

SARS-CoV-2 infection status had a substantial impact on early immune profile with prior natural infection serving to stabilise peak antibody profile following primary series vaccination although a 3^rd^ vaccine was required to limit antibody waning. In contrast, 5 vaccines were required to stabilise antibody levels within infection-naive donors.

A slightly different profile was seen in relation to cellular responses which were clearly enhanced by primary series vaccination in all donors, required 3 vaccines to reach equity in relation to infection status, and were more stable than humoral immunity. However, this representation is for information only and, as the data have not been tested by statistical analysis, causal dose thresholds and stabilization effects cannot be interpreted.

Spearman’s correlation shows that antibody levels in prior infected donors are stable from study onset whilst other measures increase over time: Antibody response PI: r = 0.38 p = 0.12; NPI: r = 0.62 p = 0.005. Spike specific cellular response PI: r = 0.66 p = 0.015 NPI: r = 0.64 p = 0.022.

### SARS-CoV-2 reinfection is more common in LTCF residents but does not increase spike-specific antibody titre

We next assessed prevalence of SARS-CoV-2 infection and reinfection by comparison of nucleocapsid-specific antibody responses in matched participant samples from July 2022 to January 2023 (median interval of 105 days, IQR 99−129 days) when donors had received at least 3 SARS-CoV-2 vaccines. A 2-fold increase in nucleocapsid-specific antibody titre was taken to reflect primary infection or reinfection (S2 Fig) although in the absence of viral screening it was not formally possible to distinquish between new infection, late seroconversion, or potential boosting from recent viral exposure.

**Fig 6:**
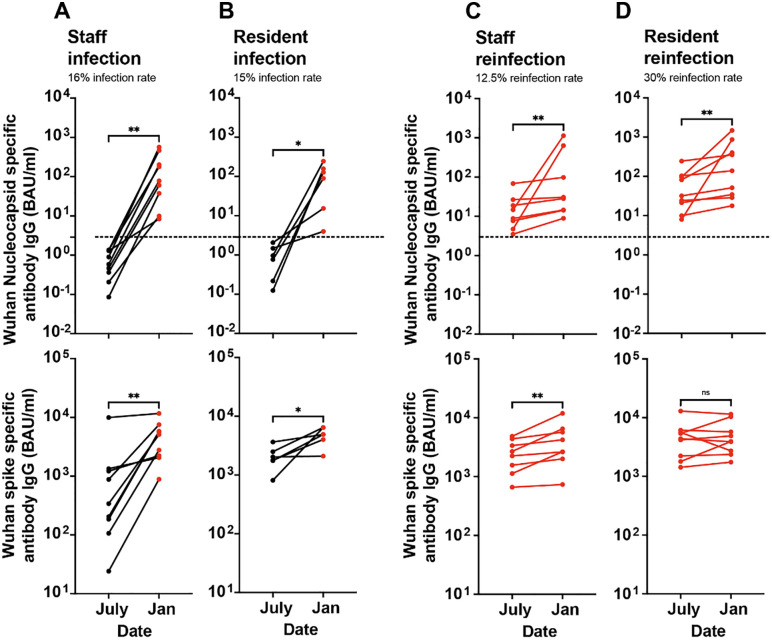
High rates of primary and secondary SARS-CoV-2 infection within LTCF. A. Nucleocapsid-specific antibody titre from seroconverted staff members. Lines indicate matched samples. Wilcoxon matched-pairs signed rank test. **p = 0.003 n = 9. Spike specific antibody titre. Wilcoxon matched-pairs signed rank test. **p = 0.003 n = 9. B. Nucleocapsid-specific antibody titre from seroconverted residents. Lines indicate matched samples. Wilcoxon matched-pairs signed rank test. *p = 0.032 n = 6. Spike specific antibody titre. Wilcoxon matched-pairs signed rank test. *p = 0.031 n = 6. C. Nucleocapsid-specific antibody titre from reinfected staff. Lines indicate matched samples. Wilcoxon matched-pairs signed rank test. **p = 0.003 n = 9. Spike specific antibody titre. Wilcoxon matched-pairs signed rank test. p = 0.91 n = 9. D. Nucleocapsid-specific antibody titre from reinfected residents. Lines indicate matched samples. Wilcoxon matched-pairs signed rank test. **p = 0.007 n = 8. Spike specific antibody titre. Wilcoxon matched-pairs signed rank test. ** p = 0.007 n = 8.

16% of staff and 15% of residents developed a primary infection whilst reinfection rates were 13% and 30% respectively. Primary infection boosted spike-specific antibody responses whilst these were not enhanced following reinfection in residents, likely reflecting a ceiling/ plateau effect or stable hybrid immunity, although a modest increase was seen in staff (Fig 6).

The results indicate continuing high rates of primary and secondary infection within the LTCF environment despite strong spike-specific immunity.

## Discussion

SARS-CoV-2 vaccines provide the cornerstone of clinical protection from Covid-19 and their immunogenicity and clinical utility in the residential care home setting is important to assess. Our findings in this large prospective national study offer confidence that the current regime of serial ancestral and Omicron-specific vaccines elicits robust and stable adaptive immune responses against SAR-CoV-2. Despite this, infection and reinfection rates remain high indicating a potential requirement for adjunctive protective approaches.

Spike-specific antibodies are an established correlate of protection and it augurs well that antibody waning was largely overcome following 5th vaccine, as seen in previous reports [14,15]. Longer term follow-up will be required to assess this further [16,17]. Bivalent vaccination also enhanced antibody responses against BA.2 and BA.5 variants, in line within pivotal early studies in older adults [18]. Antibody neutralisation was also markedly increased and supports evidence for the utility of mRNA bivalent products to overcome immune imprinting [19,20]. These incremental responses may underpin the improvement in clinical protection seen from bivalent vaccines in older people within national studies [21].

Cellular immune responses are critical for clinical protection and resilient responses were seen after a 5th vaccine, irrespective of infection status. It is typically reported that cellular responses are largely insensitive to Omicron mutations, and it was therefore interesting to see that recognition of the BA.5 variant was modestly reduced although the small sample size in infection-naive donors limits the precision of these estimates. However, a similar profile has been seen in recipients of solid organ transplants [22] and will require careful monitoring if mutation acquisition were to become concentrated within immunodominant spike peptide epitopes [23].

The fact that we had undertaken long term prospective studies of SARS-CoV-2-specific immunity across the vaccine rollout in the LTCF setting allowed us to undertake a temporal analysis of humoral and cellular responses. A striking feature was the major impact that natural infection had on the magnitude and stability of immune responses. Older residents who survived natural infection have been shown to develop robust virus-specific immunity [10] and hybrid immunity following vaccination was profound. In contrast, infection-naive donors require 5 vaccines to achieve comparable responses. Most people have now had at least one SARS-CoV-2 infection, and this latter group may therefore become largely of historical interest only. The findings demonstrate the rationale for a serial vaccine delivery regime and may underpin the sustained long-term protection against hospitalisation in older people [24]. One of the advantages of our study is that primary series vaccination included both mRNA or adenovirus-based primary regimens and no differences were observed in subsequent response to booster vaccine, in line with data from younger adults [25].

Although vaccines are highly effective in prevention of severe disease and death, they are less effective at preventing re-infection. Indeed, it was notable within this study that although spike-specific immunity was robust after serial vaccination, there was still a 15% primary infection rate during follow up. Re-infection rates were also high at 13% in staff and 30% in residents, although the BA.5 variant was dominant in this period and its effective immune evasion was reflected in national infection prevalence of ~4% per month. It is interesting to reflect on why robust systemic spike-specific adaptive immunity does not provide sterilising immunity against reinfection. Potential factors here could include suboptimal immune protection at mucosal sites, an immune ‘ceiling’ effect that is not able to overcome rapid local increase in viral load, or immune evasion of the BA.5 variant.

Nevertheless, this frequency of re-infection in the vulnerable resident population is of concern and has been seen in other settings [26]. This may reflect intrinsic vulnerabilities within the LTCF setting, including close-knit living conditions, shared amenities, and prevalent comorbidities [27]. A limitation of our study is that the clinical significance of these infections and reinfections was not determined. As such, these episodes may be of modest importance if clinically mild. However, as immunity wanes we may start to see reinfections that are associated with severe outcome, hospitalisation or death and it will be important to monitor the incidence and severity of such infections over time. The development of interventions or therapeutic approaches that can reduce reinfection risk could still remain of considerable importance. Potential options here include behavioural approaches such as sanitation or infection isolation, as well as approaches to broaden immune defence through strengthening protection against the spike protein of circulating viral variants or boosting immunity against other viral proteins.

Our study has a number of limitations. A significant one is the modest sample size for some analyses and limited pairing of samples across analyses, which reflects limitation in sample size collection from care home residents. These include the lack of routine and case-specific virological testing or information on clinical severity of infection. Demographic information is also limited in relation to determinants such as ethnicity, comorbidities, frailty, or medication use. Further, antibody responses against nucleocapsid are known to wane more rapidly than those against spike protein and, as such, it is conceivable that some donors that were regarded as non-infected had suffered a prior infection.

In summary, humoral and cellular spike-specific responses in residents of long-term care facilities plateau after 5 vaccine doses but, despite this, infection and reinfection rates remain high. This indicates an area of residual clinical unmet need which would benefit from additional preventative measures.

## Supporting information

S1 Fig Bivalent vaccine increases BA.1 specific virus neutralisation in LTCF residents but not against current circulating VOCs. A. Antibody neutralisation of ancestral (Wuhan), BA.1 XBB.1.16 and XBB1.15 variants from LTCF residents with no prior infection in relation to time after 4th (6–100 and >100 days) and 5th (6–100 and >100 days) vaccine. Top and bottom dotted lines indicate the continuous region of the data obtained. Between the bottom and one higher region is the weak inhibition region The area between the top two lines is the complete inhibition region. 2 way ANOVA (Tukey’s multiple comparisons test), *p  =  0.013, **p  =  0.005, n  =  40. B. Antibody neutralisation of ancestral (Wuhan), BA.1 XBB.1.16 and XBB1.15 variants from care home residents with prior infection shown in relation to time after 4th (6–100 and >100 days) and 5th (6–100 and >100 days) vaccines. Top and bottom dotted lines indicate the continuous region of the data obtained. Between the bottom and one higher region is the weak inhibition region The area between the top two lines is the complete inhibition region. 2 way ANOVA (Tukey’s multiple comparisons test), *p  =  0.013, **p  =  0.005, p=<0.0001 n  =  92.
(DOCX)

S2 Fig Anti nucleocapsid and spike specific antibody levels in LTCF residents and staff members that did not change infection status. A. Nucleocapsid specific antibody titre from LTCF residents and staff at two collections. Lines indicate matched samples. Paired T test (Wilcoxon) ****p=<0.0001 n = 64. B. Nucleocapsid specific antibody IgG titre changes between samples collected in July vs Jan between prior infected (PI, red) and non prior infected(NPI, black). n = 64, T-test, ***p = 0.0002. C. Spike specific antibody titre from LTCF residents and staff at two collections. Lines indicate matched samples. Paired T test (Wilcoxon) no statistical difference seen.
(DOCX)
