# Supplementary material for: High rates of SARS-CoV-2 reinfection in residents of long term care facilities despite robust spike-specific immunity following serial vaccination
Source: PLoS One. 2026 Aug 14;21(8):e0354079. doi: 10.1371/journal.pone.0354079 (PMC13475887; doi:10.1371/journal.pone.0354079)
Supplement: Supplementary file 3 [file pone.0354079.s004.docx]

**PONE-D-25-51786
High rates of SARS-CoV-2 reinfection in residents of long term care facilities despite robust spike-specific immunity following serial vaccination.**

**Response to Reviewers**

**We thank the reviewer for their very positive and incisive assessment of our manuscript. We make details comments to each point below.**

Reviewer #1: This manuscript by Tut & Lancaster et al. presents a valuable investigation into the immune response and clinical outcomes following serial SARS-CoV-2 vaccination, including a bivalent booster, in the highly vulnerable nursing home population. The study leverages the VIVALDI cohort to provide longitudinal data on both humoral and cellular immunity. The central finding that robust, plateaued spike-specific immunity does not prevent high rates of reinfection is clinically important. However, I hope the authors would find the comments below helpful in improving the manuscript.

***We thank the Reviewer for these positive and helpful comments.***

Minor
The final sentence in the abstract is strong, but the preceding one ("These findings demonstrate...support the value of bivalent vaccines") feels somewhat generic. Consider refining it to more directly reflect the nuanced finding that the vaccine boosts immunity but that this alone is insufficient to prevent infection in this setting.

***Thank you. This has now been done.***

Line 71 and 72 are duplicates (methods)

***We apologise this has been corrected.***

The relationship between the 4th and 5th doses and sample collection needs clarification. Lines 124-127 show a much longer median time between the 4th dose and sampling for residents (133 days) compared to the 5th dose (65 days). This complicates the direct comparison of immune responses between the 4th and 5th doses, as waning may have already occurred for the 4th dose group. This could be acknowledged as a limitation.

***Thank you. This has now been listed as a limitation and is discussed in the results section.***

Clarify the role of staff participants. Their inclusion is appropriate as a comparison group, but the manuscript could explain why only three staff received the 5th dose and how their data were handled.

***This is a very reasonable point and is now explicitly addressed in the results section***

The introduction references “more reassuring outcomes observed in other settings (refs 4,5)” but does not clearly contrast those findings with the current study.

***We apologise for this and these manuscripts have now been described more fully in the Introduction.***

There are important variables that are missing or briefly mentioned which greatly influence immune response in this population. While missing variable such as ethnicity, time since prior infection, severity of infections or reinfections were acknowledged, others such as comorbidities, frailty, or medication use need be acknowledged.

***Thank you for identifying this limitation which has now been discussed.***


Major comments
- Reinfection is defined solely by a ≥2-fold rise in nucleocapsid IgG. This threshold is not validated, especially in older adults, and natural fluctuations or assay variability could account for such changes. Also, the text acknowledges reduced PCR/LFD testing in later periods, yet the manuscript does not quantify how this limited diagnostic certainty may bias reinfection estimates. Without sequencing or precise timing of infection, it is unclear whether rises in nucleocapsid antibody reflect new infection, late seroconversion, or boosting from recent viral exposure. Authors should justify the two-fold threshold using published references or internal validation. Also consider discussing the risk of misclassification more explicitly.

***Thank you for this important point. We agree that in the absence of routine PCR/LFD testing it can be challenging to define coronavirus reinfection and serological assessment must be performed. We utilised a ≥2-fold rise in nucleocapsid IgG as we utilise the MSD platform for antibody measurement and this gives accurate quantification, considerably better than ELISA assays, and we have previously found this of value (PMID: 37391077). Nucleocapsid-specific titres are also notable for waning, with typical t_1/2_ of 3-6 months, and so any increase above baseline value is highly suggestive of repeat antigen exposure. Nevertheless, the reviewer is entirely correct that this was not validated in this cohort and it is not possible to distinquish between new infection, late seroconversion, or boosting from recent viral exposure. As such, this has now been fully discussed in the Results and Discussion section.***

- A major limitation, acknowledged by the authors, is the lack of data on the clinical severity of these primary infections and reinfections. Given that the primary goal of vaccination in this population is to prevent severe disease, hospitalization, and death, the absence of this data significantly weakens the public health message. Were these largely asymptomatic infections detected by routine screening? Or did they lead to hospitalizations? Any available data on symptom severity or healthcare utilization should be included. If no data exists, this key limitation needs to be more explicitly stated. It may be helpful to clarify that reinfection rates may reflect environmental/behavioral exposure rather than vaccine insufficiency. Also, I would avoid implying that reinfections necessarily indicate unmet clinical need unless clinical significance is demonstrated.

***This is a very important point and we appreciate that the clinical severity of SARS-CoV-2 reinfection is likely to decrease over time due to accumulation of protective immunity. We have now added text to further discuss this and to bring out the fact that the clinical importance of approaches that reduce reinfection rates must be constantly reassessed and related to populations at particular risk.***

- The reported 30% reinfection rate in residents over 6 months is presented as "high," which it may well be. However, to properly interpret this, the manuscript needs context. What was the community incidence of SARS-CoV-2 during this specific study period (approx. July 2022 - Jan 2023)? This period likely covered Omicron subvariants like BA.5, which are known for high immune evasion. Stating the dominant variants and community prevalence would help assess whether this 30% rate is truly unexpectedly high given the immune-evasive nature of the circulating viruses and the congregate living setting, or if it is consistent with broader population trends.

***We agree that the use of the word ‘high’ is somewhat subjective. We have now included the important information that BA.5 was highly infectious and background national prevalence was high***

- The data in Figure 6 and the corresponding text (lines 340-342) are currently confusing. It states that reinfection did not boost spike-specific antibodies in residents "due to high baseline values." However, Figure 6D appears to show a significant increase in spike antibodies in reinfected residents (p=0.007). This direct contradiction between the text and the figure must be resolved. The authors need to clarify their interpretation and ensure the statistical analysis and textual description are aligned.

***Thank you for this. In fact, the antibody increase after reinfection in residents is seen only in the nucleocapsid-specific response. However, there is a statistical increase in spike-specific titre in staff and this has been added to the text. We apologise for the confusion.***

- The discussion focuses on the robustness of the immune response but does not adequately grapple with the core paradox: why do high antibody and T-cell levels not prevent infection? The authors correctly state that vaccines are less effective at preventing infection than severe disease, but they could expatiate further. For instance: Could this be due to differences in mucosal IgA responses, which are critical for blocking infection at the portal of entry? Could the "plateau" of immunity represent a ceiling effect that is still insufficient to neutralize the high viral loads encountered in an LTCF setting? The modest reduction in BA.5-specific T-cell responses is noted, but its potential contribution to breakthrough infections could be discussed more thoroughly.

***These are wise points and we have added this discussion to the text.***

- The suggestion that "further approaches are needed" is appropriate but vague. The discussion could be strengthened by proposing specific "adjunctive protective approaches" (line 369), such as improved ventilation, prophylactic antivirals, or perhaps, next-generation mucosal vaccines.

***Thank you. We have now added this information.***

Reviewer #2: The authours have evaluated Older adult residents of long-term care facilities, and their infection/reinfection rates with Sara-CoV-2. While the maunscript is generally acceptable for publication and the data and conculsions are justified. I have some questions that may be considered.

***Thank you for this assessment.***

The manuscript puts a large amount of emphesis on the 4th to 5th vacine dose "Here we determined humoral and cellular immunity following delivery of a 5th vaccine dose" however Table 2 tells us that only 3 staff (of 168) and 64 residents (of 141) recived the 5th dose. in total only 22% of those in the study. Is it therefore correct to put such emphesis on the 4th to 5th vaccine dose when only 67 people of the 309 in the study actually recived this dose?. Also the inclusion of the Staff - who are a wholely different demographic - mostly much much younger than the residents - this also seems like an odd sub set to be included in the study - which doesnt really get an explanation.

***Thank you. Very few staff received a 5^th^ vaccine as they were generally much younger and were therefore not in the demographic group to receive additional booster vaccines. We have now discussed this limitation in the text.***

The manuscript could also do with a quick grammar check.

***Thank you. We have now reviewed the complete text and make it more accessible to readers.***
