# Supplementary material for: High rates of SARS-CoV-2 reinfection in residents of long term care facilities despite robust spike-specific immunity following serial vaccination
Source: PLoS One. 2026 Aug 14;21(8):e0354079. doi: 10.1371/journal.pone.0354079 (PMC13475887; doi:10.1371/journal.pone.0354079)
Supplement: Supplementary file 4 [file pone.0354079.s005.docx]

**PONE-D-25-51786**

**Second response to Reviewers**

**Comments to the Author**

1. If the authors have adequately addressed your comments raised in a previous round of review and you feel that this manuscript is now acceptable for publication, you may indicate that here to bypass the “Comments to the Author” section, enter your conflict of interest statement in the “Confidential to Editor” section, and submit your "Accept" recommendation.

Reviewer #1: All comments have been addressed

Reviewer #3: (No Response)

2. Is the manuscript technically sound, and do the data support the conclusions?

The manuscript must describe a technically sound piece of scientific research with data that supports the conclusions. Experiments must have been conducted rigorously, with appropriate controls, replication, and sample sizes. The conclusions must be drawn appropriately based on the data presented.

Reviewer #1: Yes

Reviewer #3: Yes

3. Has the statistical analysis been performed appropriately and rigorously?

Reviewer #1: Yes

Reviewer #3: No

4. Have the authors made all data underlying the findings in their manuscript fully available?

The [PLOS Data policy](https://urldefense.com/v3/__http:/track.editorialmanager.com/CL0/http:*2F*2Fwww.plosone.org*2Fstatic*2Fpolicies.action*23sharing/1/010f019dfbdc2cb9-400f1e69-97bd-4943-a048-553d05850a7a-000000/LdhFQHT5TKnPB-69BuicVMYIkGrKOH0uIsXAH5aWKPk=258__;JSUlJSU!!CF15FET90Tp8!FE-VeShK5R89l2qE50FEnTqID5Jvp7EU-0PEj_UWbULAUIv6Ft4D07DthVgUl2YpGRmm2QdmynzW2gBQlg$) requires authors to make all data underlying the findings described in their manuscript fully available without restriction, with rare exception (please refer to the Data Availability Statement in the manuscript PDF file). The data should be provided as part of the manuscript or its supporting information, or deposited to a public repository. For example, in addition to summary statistics, the data points behind means, medians and variance measures should be available. If there are restrictions on publicly sharing data—e.g. participant privacy or use of data from a third party—those must be specified.

Reviewer #1: (No Response)

Reviewer #3: Yes

5. Is the manuscript presented in an intelligible fashion and written in standard English?

PLOS ONE does not copyedit accepted manuscripts, so the language in submitted articles must be clear, correct, and unambiguous. Any typographical or grammatical errors should be corrected at revision, so please note any specific errors here.

Reviewer #1: Yes

Reviewer #3: Yes

6. Review Comments to the Author

Please use the space provided to explain your answers to the questions above. You may also include additional comments for the author, including concerns about dual publication, research ethics, or publication ethics. (Please upload your review as an attachment if it exceeds 20,000 characters)

Reviewer #1: (No Response)

Reviewer #3: The aim of this study was to 1. Determine the antibody and cellular immune responses against SARS-CoV-2 following bivalent vaccination in long-term care facilities for older adults, and to 2. Assess breakthrough infection rates. The study uses a subset of VIVALDI cohort to compare immune responses after later booster doses (4th and 5th) to conclude that while the vaccines elicit robust systemic spike-specific immune responses, it does not prevent reinfection. The manuscript addresses an epidemiologically important question and has important public health implications for vulnerable older populations. However, the authors should consider these revisions to allow accurate interpretation of the data.

**Thank you for advising on these potential revisions. We have undertaken these and have made substantial statistical revisions throughout the paper. These have further improved the manuscript and we thank the reviewer for the advice.**

• It is unclear which participants were included in the analyses and in each figure. While the table nicely breaks down the population in the study, it doesn’t provide information about which samples actually ended up in the analyses and the breakdown of staff vs residents. For example, Figure 1A reports an n=41, are these all residents? Are these completely independent samples? Or are there repeated participants in each group? Similarly, Figures 4 and 6 have a different sample size compared to other figures with matched data. The manuscript can really benefit from mapping the included participants/samples into a CONSORT-style diagram and from noting when certain samples are omitted from analysis in specific figures. The sample sizes in matched data are very small. Why?

**Thank you for this. The study was undertaken using small sample collections from frail residents in care homes and sometimes not enough cells or plasma was available for study. Laboratory analysis was also undertaken on two sites to maximise expertise. However, we have now made the limitation on sample size and pairing the major focus in our section on the limitations of the study in the Discussion section.**

• Statistical analyses in all figures need reconsideration. In figure 1A and C, it is not clear if the analysis included repeated participants (i.e. same people contributed a sample after the 4th dose and after 5th dose). If participants are repeated, then the 4 groups are not fully independent so Kruskal–Wallis test is not appropriate, and a mixed-effects model should be used.

**Thank you for this valuable advice.**

**A mixed effects model has been applied to Figures 1A and 1C. A new figure has been replaced in this regard. The figure legends have been amended appropriately. This analysis removes a prior statistical difference between the groups. However, the PI model showed that the Spike over time was significantly different with a statistical significance in early 4^th^ and later 5^th^ and early 5^th^ and late 5^th^ groups.**

• In panels B and D, it would be nice to see a correlation graph between antibody titer after 4th and 5th dose instead of showing only matched data. Also, when were these matched samples collected for the 4th and 5th dose? Are equivalent time points being compared?
• 6–100 days is a very wide window. Immune responses at one week and at 3 months are not biologically equivalent, and you lose a lot of information. I suggest further stratifying the groups.

**Thank you for this advice. It is indeed a wide window and this has been added to the limitations of the manuscript.**

• Figure 2: Why were the Wuhan titers for prior infection samples analyzed with paired t test and the rest of the titers analyzed with Wilcoxon matched-pairs signed rank test? Were multiple comparisons done and corrected? Or were these unadjusted comparisons? The authors need to reanalyze and draw conclusions accordingly. From the current p values, bivalent vaccination broadened Omicron spike-binding responses more consistently in prior-infected than in infection-naïve participants. Also, similar to Figure 1, it should be stated whether the paired samples were taken at comparable times after each dose.

**Thank you for this. Wilcoxon matched-pairs has been performed on the figure.**

**Comparisons were restricted to five pre-specified SARS-CoV-2 variants selected a priori for their biological relevance. These analyses were hypothesis-driven rather than exploratory, with each comparison addressing a predefined research question rather than representing a broad, data-driven search for associations. Consequently, we did not apply a multiple-testing correction, as our understanding is that such adjustments are generally intended to control the false-positive rate in exploratory analyses involving large numbers of simultaneous comparisons and may be unnecessarily conservative for such a small set of pre-specified hypotheses.**

• Figure 3: again, statistical analysis. Were multiple comparisons done? What were the p values for each variant? How were the thresholds for weak and complete inhibition determined? Or are these the limits of detection of the assay? How did the authors reach the conclusion “bivalent vaccine enhances omicron variant-specific antibody titres irrespective of infection status and further enhances neutralisation capacity in donors who remain infection-naive”?

**Thank you.**

**Thresholds for weak and complete inhibition were determined by manufacturers’ guidance. Comparisons were restricted to five pre-specified SARS-CoV-2 variants selected a priori for their biological relevance. These analyses were hypothesis-driven rather than exploratory, with each comparison addressing a predefined research question rather than representing a broad, data-driven search for associations.**

**Consequently, we did not apply a multiple-testing correction, as such adjustments are generally intended to control the false-positive rate in exploratory analyses involving large numbers of simultaneous comparisons and may be unnecessarily conservative for such a small set of pre-specified hypotheses.**

**Wilcoxon matched-pairs has now been performed on the figure**

• Figure 4: The manuscript concludes that “Recognition of ancestral and BA.1 peptides was equivalent whilst those against BA.5 were slightly reduced, irrespective of prior infection status”. While cellular responses to BA.5 were modestly reduced in both prior-infected and infection-naive residents, the small sample size in infection-naive donors limits the precision of these estimates. Therefore, conclusions should be framed cautiously.

**Thank you for spotting this. We agree that we over interpreted this effect. This has been rewritten accordingly.**

• Figure 5 is informative, but authors should be cautious about overstatements and implying causal dose thresholds and stabilization effects that are not directly tested by the statistical analysis presented. To evaluate changes in immune responses across doses and infection status groups, a mixed-effects longitudinal model would be more appropriate. Additionally, if this data is from all participants in the VIVALDI study, does that mean all ages are included?

**Thank you. We have now amended the text to say that. “However, this representation is for information only and, as the data have not been tested by statistical analysis, causal dose thresholds and stabilization effects cannot be interpreted”.**

• Figure 6: How many doses of vaccine did the participants in this figure have at that point? While Primary infection and reinfection events occurred, it is unclear how it was determined that the primary infection participants had “strong immunity”. Some of these participants were sitting at 10–100 BAU/mL in July, that is a low binding-antibody level. The data suggest that a subset of older donors remained weak humoral responders or had substantial waning in July, but with infection, immune responses were boosted. Overall, immunity in this cohort was shaped by both vaccination and repeated infection. In the resident reinfection group, infection did not result in further measurable boosting of spike antibody levels, likely reflecting a ceiling/ plateau effect or stable hybrid immunity.

**Thank you. Recipients had received at least 3 vaccines. We have rewritten this section based on the reviewers comments.**

Minor concerns:
• In addition to the absence of viral screening and inability to tell the time of prior infection with the use of anti-N IgG, it would strengthen the manuscript to acknowledge that anti-N IgGs decline more rapidly than spike antibodies, especially in older adults. Therefore, there could be some misclassification in the “naive group”.

**Thank you. This is an important point and this has been more clearly stated in the limitations section.**

• Additional clarification and description of the analytic structure and interpretation of results in the Results section would substantially improve readability and reduce ambiguity.

**Thank you. This has been added to the Methods section.**

• Line 343, the text says August 2022 but the figure says July. Make sure it is consistent.

**We apologise for this error which has been corrected.**
