# Supplementary material for: High rates of SARS-CoV-2 reinfection in residents of long term care facilities despite robust spike-specific immunity following serial vaccination
Source: PLoS One. 2026 Aug 14;21(8):e0354079. doi: 10.1371/journal.pone.0354079 (PMC13475887; doi:10.1371/journal.pone.0354079)
Supplement: Supplementary file 5 [file pone.0354079.s001.docx]

**Supporting Information**

**Supplementary Figure 1**
