# Supplementary material for: High rates of SARS-CoV-2 reinfection in residents of long term care facilities despite robust spike-specific immunity following serial vaccination
Source: PLoS One. 2026 Aug 14;21(8):e0354079. doi: 10.1371/journal.pone.0354079 (PMC13475887; doi:10.1371/journal.pone.0354079)
Supplement: Peer review history

# Author comment: Author response to Decision Letter 0

**DOI:** 10.1371/journal.pone.0354079.r001

**Transfer Alert**

This paper was transferred from another journal. As a result, its full editorial history (including decision letters, peer reviews and author responses) may not be present.

21 Oct 2025

# Peer review: Decision Letter 0

**DOI:** 10.1371/journal.pone.0354079.r002

**Authors:** Dhammika Leshan Wannigama

30 Dec 2025

Dear Dr. Moss,

Thank you for submitting your manuscript to PLOS ONE. After careful consideration, we feel that it has merit but does not fully meet PLOS ONE’s publication criteria as it currently stands. Therefore, we invite you to submit a revised version of the manuscript that addresses the points raised during the review process.

Please submit your revised manuscript by Feb 13 2026 11:59PM. If you will need more time than this to complete your revisions, please reply to this message or contact the journal office at plosone@plos.org. When you're ready to submit your revision, log on to https://www.editorialmanager.com/pone/ and select the 'Submissions Needing Revision' folder to locate your manuscript file.

- A letter that responds to each point raised by the academic editor and reviewer(s). You should upload this letter as a separate file labeled 'Response to Reviewers'.
- A marked-up copy of your manuscript that highlights changes made to the original version. You should upload this as a separate file labeled 'Revised Manuscript with Track Changes'.
- An unmarked version of your revised paper without tracked changes. You should upload this as a separate file labeled 'Manuscript'.

If you would like to make changes to your financial disclosure, please include your updated statement in your cover letter. Guidelines for resubmitting your figure files are available below the reviewer comments at the end of this letter.

If applicable, we recommend that you deposit your laboratory protocols in protocols.io to enhance the reproducibility of your results. Protocols.io assigns your protocol its own identifier (DOI) so that it can be cited independently in the future. For instructions see: https://journals.plos.org/plosone/s/submission-guidelines#loc-laboratory-protocols. Additionally, PLOS ONE offers an option for publishing peer-reviewed Lab Protocol articles, which describe protocols hosted on protocols.io. Read more information on sharing protocols at https://plos.org/protocols?utm_medium=editorial-email&utm_source=authorletters&utm_campaign=protocols.

We look forward to receiving your revised manuscript.

Kind regards,

Dhammika Leshan Wannigama, MD PhD

Academic Editor

PLOS One

Journal Requirements:

When submitting your revision, we need you to address these additional requirements.

1.Please ensure that your manuscript meets PLOS ONE's style requirements, including those for file naming. The PLOS ONE style templates can be found at

https://journals.plos.org/plosone/s/file?id=wjVg/PLOSOne_formatting_sample_main_body.pdf and

https://journals.plos.org/plosone/s/file?id=ba62/PLOSOne_formatting_sample_title_authors_affiliations.pdf

2. Thank you for stating the following financial disclosure:

“This work was funded by the Department of Health and Social Care (COVID-19 surveillance studies). The work was supported by the Francis Crick Institute which receives its core funding from Cancer Research UK (CC2230, CC2087), the UK Medical Research Council (CC2230, CC2087) and the Wellcome Trust (CC2230, CC2087).”

Please state what role the funders took in the study.  If the funders had no role, please state: "The funders had no role in study design, data collection and analysis, decision to publish, or preparation of the manuscript."

If this statement is not correct you must amend it as needed.

Please include this amended Role of Funder statement in your cover letter; we will change the online submission form on your behalf.

3. In the online submission form you indicate that your data is not available for proprietary reasons and have provided a contact point for accessing this data. Please note that your current contact point is a co-author on this manuscript. According to our Data Policy, the contact point must not be an author on the manuscript and must be an institutional contact, ideally not an individual. Please revise your data statement to a non-author institutional point of contact, such as a data access or ethics committee, and send this to us via return email. Please also include contact information for the third party organization, and please include the full citation of where the data can be found.

4. Please amend the manuscript submission data (via Edit Submission) to include author Gokhan Tut,Tara Lancaster, Panagiota Sylla, David Bone, Christopher Bentley, Azar Jadir, Rachel Bruton, Katie Spencer, Soumyajit Mallick, Ahmed Elzaidi, Alexander Dowell,Mary Y Wu, Ruth Harvey, Edward J Carr, Crick COVID Immunity Pipeline, Rupert Beale, Maria Krutikov, Oliver Stirrup, Borscha Azmi, Andrew Hayward, Andrew Copas and Laura Shallcross.

5. Please ensure that you refer to Figure 5 and 6 in your text as, if accepted, production will need this reference to link the reader to the figure.

6. Please remove your figures from within your manuscript file, leaving only the individual TIFF/EPS image files, uploaded separately. These will be automatically included in the reviewers’ PDF.

7. We notice that your supplementary figures are included in the manuscript file. Please remove them and upload them with the file type 'Supporting Information'. Please ensure that each Supporting Information file has a legend listed in the manuscript after the references list.

8. If the reviewer comments include a recommendation to cite specific previously published works, please review and evaluate these publications to determine whether they are relevant and should be cited. There is no requirement to cite these works unless the editor has indicated otherwise.

[Note: HTML markup is below. Please do not edit.]

Reviewers' comments:

Reviewer's Responses to Questions

**Comments to the Author**

1. Is the manuscript technically sound, and do the data support the conclusions?

Reviewer #1: Yes

Reviewer #2: Yes

**********

2. Has the statistical analysis been performed appropriately and rigorously?

Reviewer #1: Yes

Reviewer #2: Yes

**********

3. Have the authors made all data underlying the findings in their manuscript fully available?

The PLOS Data policy

Reviewer #1: Yes

Reviewer #2: No

**********

4. Is the manuscript presented in an intelligible fashion and written in standard English?

Reviewer #1: Yes

Reviewer #2: Yes

**********

Reviewer #1: This manuscript by Tut & Lancaster et al. presents a valuable investigation into the immune response and clinical outcomes following serial SARS-CoV-2 vaccination, including a bivalent booster, in the highly vulnerable nursing home population. The study leverages the VIVALDI cohort to provide longitudinal data on both humoral and cellular immunity. The central finding that robust, plateaued spike-specific immunity does not prevent high rates of reinfection is clinically important. However, I hope the authors would find the comments below helpful in improving the manuscript.

Minor

The final sentence in the abstract is strong, but the preceding one ("These findings demonstrate...support the value of bivalent vaccines") feels somewhat generic. Consider refining it to more directly reflect the nuanced finding that the vaccine boosts immunity but that this alone is insufficient to prevent infection in this setting.

Line 71 and 72 are duplicates (methods)

The relationship between the 4th and 5th doses and sample collection needs clarification. Lines 124-127 show a much longer median time between the 4th dose and sampling for residents (133 days) compared to the 5th dose (65 days). This complicates the direct comparison of immune responses between the 4th and 5th doses, as waning may have already occurred for the 4th dose group. This could be acknowledged as a limitation.

Clarify the role of staff participants. Their inclusion is appropriate as a comparison group, but the manuscript could explain why only three staff received the 5th dose and how their data were handled.

The introduction references “more reassuring outcomes observed in other settings (refs 4,5)” but does not clearly contrast those findings with the current study.

There are important variables that are missing or briefly mentioned which greatly influence immune response in this population. While missing variable such as ethnicity, time since prior infection, severity of infections or reinfections were acknowledged, others such as comorbidities, frailty, or medication use need be acknowledged.

Major comments

- Reinfection is defined solely by a ≥2-fold rise in nucleocapsid IgG. This threshold is not validated, especially in older adults, and natural fluctuations or assay variability could account for such changes. Also, the text acknowledges reduced PCR/LFD testing in later periods, yet the manuscript does not quantify how this limited diagnostic certainty may bias reinfection estimates. Without sequencing or precise timing of infection, it is unclear whether rises in nucleocapsid antibody reflect new infection, late seroconversion, or boosting from recent viral exposure. Authors should justify the two-fold threshold using published references or internal validation. Also consider discussing the risk of misclassification more explicitly.

- A major limitation, acknowledged by the authors, is the lack of data on the clinical severity of these primary infections and reinfections. Given that the primary goal of vaccination in this population is to prevent severe disease, hospitalization, and death, the absence of this data significantly weakens the public health message. Were these largely asymptomatic infections detected by routine screening? Or did they lead to hospitalizations? Any available data on symptom severity or healthcare utilization should be included. If no data exists, this key limitation needs to be more explicitly stated. It may be helpful to clarify that reinfection rates may reflect environmental/behavioral exposure rather than vaccine insufficiency. Also, I would avoid implying that reinfections necessarily indicate unmet clinical need unless clinical significance is demonstrated.

- The reported 30% reinfection rate in residents over 6 months is presented as "high," which it may well be. However, to properly interpret this, the manuscript needs context. What was the community incidence of SARS-CoV-2 during this specific study period (approx. July 2022 - Jan 2023)? This period likely covered Omicron subvariants like BA.5, which are known for high immune evasion. Stating the dominant variants and community prevalence would help assess whether this 30% rate is truly unexpectedly high given the immune-evasive nature of the circulating viruses and the congregate living setting, or if it is consistent with broader population trends.

- The data in Figure 6 and the corresponding text (lines 340-342) are currently confusing. It states that reinfection did not boost spike-specific antibodies in residents "due to high baseline values." However, Figure 6D appears to show a significant increase in spike antibodies in reinfected residents (p=0.007). This direct contradiction between the text and the figure must be resolved. The authors need to clarify their interpretation and ensure the statistical analysis and textual description are aligned.

- The discussion focuses on the robustness of the immune response but does not adequately grapple with the core paradox: why do high antibody and T-cell levels not prevent infection? The authors correctly state that vaccines are less effective at preventing infection than severe disease, but they could expatiate further. For instance: Could this be due to differences in mucosal IgA responses, which are critical for blocking infection at the portal of entry? Could the "plateau" of immunity represent a ceiling effect that is still insufficient to neutralize the high viral loads encountered in an LTCF setting? The modest reduction in BA.5-specific T-cell responses is noted, but its potential contribution to breakthrough infections could be discussed more thoroughly.

- The suggestion that "further approaches are needed" is appropriate but vague. The discussion could be strengthened by proposing specific "adjunctive protective approaches" (line 369), such as improved ventilation, prophylactic antivirals, or perhaps, next-generation mucosal vaccines.

Reviewer #2: The authours have evaluated Older adult residents of long-term care facilities, and their infection/reinfection rates with Sara-CoV-2. While the maunscript is generally acceptable for publication and the data and conculsions are justified. I have some questions that may be considered.

The manuscript puts a large amount of emphesis on the 4th to 5th vacine dose "Here we determined humoral and cellular immunity following delivery of a 5th vaccine dose" however Table 2 tells us that only 3 staff (of 168) and 64 residents (of 141) recived the 5th dose. in total only 22% of those in the study. Is it therefore correct to put such emphesis on the 4th to 5th vaccine dose when only 67 people of the 309 in the study actually recived this dose?. Also the inclusion of the Staff - who are a wholely different demographic - mostly much much younger than the residents - this also seems like an odd sub set to be included in the study - which doesnt really get an explanation.

The manuscript could also do with a quick grammar check.

**********

what does this mean?). If published, this will include your full peer review and any attached files.

If you choose “no”, your identity will remain anonymous but your review may still be made public.

**Do you want your identity to be public for this peer review?**  For information about this choice, including consent withdrawal, please see our Privacy Policy

Reviewer #1: **Yes:** Oladayo A. Oyebanji

Reviewer #2: No

**********

[NOTE: If reviewer comments were submitted as an attachment file, they will be attached to this email and accessible via the submission site. Please log into your account, locate the manuscript record, and check for the action link "View Attachments". If this link does not appear, there are no attachment files.]

To ensure your figures meet our technical requirements, please review our figure guidelines: https://journals.plos.org/plosone/s/figures

You may also use PLOS’s free figure tool, NAAS, to help you prepare publication quality figures: https://journals.plos.org/plosone/s/figures#loc-tools-for-figure-preparation.

NAAS will assess whether your figures meet our technical requirements by comparing each figure against our figure specifications.

# Author comment: Author response to Decision Letter 1

**DOI:** 10.1371/journal.pone.0354079.r003

24 Mar 2026

PONE-D-25-51786

High rates of SARS-CoV-2 reinfection in residents of long term care facilities despite robust spike-specific immunity following serial vaccination.

Response to Reviewers

We thank the reviewer for their very positive and incisive assessment of our manuscript. We make details comments to each point below.

Reviewer #1: This manuscript by Tut & Lancaster et al. presents a valuable investigation into the immune response and clinical outcomes following serial SARS-CoV-2 vaccination, including a bivalent booster, in the highly vulnerable nursing home population. The study leverages the VIVALDI cohort to provide longitudinal data on both humoral and cellular immunity. The central finding that robust, plateaued spike-specific immunity does not prevent high rates of reinfection is clinically important. However, I hope the authors would find the comments below helpful in improving the manuscript.

We thank the Reviewer for these positive and helpful comments.

Minor

The final sentence in the abstract is strong, but the preceding one ("These findings demonstrate...support the value of bivalent vaccines") feels somewhat generic. Consider refining it to more directly reflect the nuanced finding that the vaccine boosts immunity but that this alone is insufficient to prevent infection in this setting.

Thank you. This has now been done.

Line 71 and 72 are duplicates (methods)

We apologise this has been corrected.

The relationship between the 4th and 5th doses and sample collection needs clarification. Lines 124-127 show a much longer median time between the 4th dose and sampling for residents (133 days) compared to the 5th dose (65 days). This complicates the direct comparison of immune responses between the 4th and 5th doses, as waning may have already occurred for the 4th dose group. This could be acknowledged as a limitation.

Thank you. This has now been listed as a limitation and is discussed in the results section.

Clarify the role of staff participants. Their inclusion is appropriate as a comparison group, but the manuscript could explain why only three staff received the 5th dose and how their data were handled.

This is a very reasonable point and is now explicitly addressed in the results section

The introduction references “more reassuring outcomes observed in other settings (refs 4,5)” but does not clearly contrast those findings with the current study.

We apologise for this and these manuscripts have now been described more fully in the Introduction.

There are important variables that are missing or briefly mentioned which greatly influence immune response in this population. While missing variable such as ethnicity, time since prior infection, severity of infections or reinfections were acknowledged, others such as comorbidities, frailty, or medication use need be acknowledged.

Thank you for identifying this limitation which has now been discussed.

Major comments

- Reinfection is defined solely by a ≥2-fold rise in nucleocapsid IgG. This threshold is not validated, especially in older adults, and natural fluctuations or assay variability could account for such changes. Also, the text acknowledges reduced PCR/LFD testing in later periods, yet the manuscript does not quantify how this limited diagnostic certainty may bias reinfection estimates. Without sequencing or precise timing of infection, it is unclear whether rises in nucleocapsid antibody reflect new infection, late seroconversion, or boosting from recent viral exposure. Authors should justify the two-fold threshold using published references or internal validation. Also consider discussing the risk of misclassification more explicitly.

Thank you for this important point. We agree that in the absence of routine PCR/LFD testing it can be challenging to define coronavirus reinfection and serological assessment must be performed. We utilised a ≥2-fold rise in nucleocapsid IgG as we utilise the MSD platform for antibody measurement and this gives accurate quantification, considerably better than ELISA assays, and we have previously found this of value (PMID: 37391077). Nucleocapsid-specific titres are also notable for waning, with typical t1/2 of 3-6 months, and so any increase above baseline value is highly suggestive of repeat antigen exposure. Nevertheless, the reviewer is entirely correct that this was not validated in this cohort and it is not possible to distinquish between new infection, late seroconversion, or boosting from recent viral exposure. As such, this has now been fully discussed in the Results and Discussion section.

- A major limitation, acknowledged by the authors, is the lack of data on the clinical severity of these primary infections and reinfections. Given that the primary goal of vaccination in this population is to prevent severe disease, hospitalization, and death, the absence of this data significantly weakens the public health message. Were these largely asymptomatic infections detected by routine screening? Or did they lead to hospitalizations? Any available data on symptom severity or healthcare utilization should be included. If no data exists, this key limitation needs to be more explicitly stated. It may be helpful to clarify that reinfection rates may reflect environmental/behavioral exposure rather than vaccine insufficiency. Also, I would avoid implying that reinfections necessarily indicate unmet clinical need unless clinical significance is demonstrated.

This is a very important point and we appreciate that the clinical severity of SARS-CoV-2 reinfection is likely to decrease over time due to accumulation of protective immunity. We have now added text to further discuss this and to bring out the fact that the clinical importance of approaches that reduce reinfection rates must be constantly reassessed and related to populations at particular risk.

- The reported 30% reinfection rate in residents over 6 months is presented as "high," which it may well be. However, to properly interpret this, the manuscript needs context. What was the community incidence of SARS-CoV-2 during this specific study period (approx. July 2022 - Jan 2023)? This period likely covered Omicron subvariants like BA.5, which are known for high immune evasion. Stating the dominant variants and community prevalence would help assess whether this 30% rate is truly unexpectedly high given the immune-evasive nature of the circulating viruses and the congregate living setting, or if it is consistent with broader population trends.

We agree that the use of the word ‘high’ is somewhat subjective. We have now included the important information that BA.5 was highly infectious and background national prevalence was high

- The data in Figure 6 and the corresponding text (lines 340-342) are currently confusing. It states that reinfection did not boost spike-specific antibodies in residents "due to high baseline values." However, Figure 6D appears to show a significant increase in spike antibodies in reinfected residents (p=0.007). This direct contradiction between the text and the figure must be resolved. The authors need to clarify their interpretation and ensure the statistical analysis and textual description are aligned.

Thank you for this. In fact, the antibody increase after reinfection in residents is seen only in the nucleocapsid-specific response. However, there is a statistical increase in spike-specific titre in staff and this has been added to the text. We apologise for the confusion.

- The discussion focuses on the robustness of the immune response but does not adequately grapple with the core paradox: why do high antibody and T-cell levels not prevent infection? The authors correctly state that vaccines are less effective at preventing infection than severe disease, but they could expatiate further. For instance: Could this be due to differences in mucosal IgA responses, which are critical for blocking infection at the portal of entry? Could the "plateau" of immunity represent a ceiling effect that is still insufficient to neutralize the high viral loads encountered in an LTCF setting? The modest reduction in BA.5-specific T-cell responses is noted, but its potential contribution to breakthrough infections could be discussed more thoroughly.

These are wise points and we have added this discussion to the text.

- The suggestion that "further approaches are needed" is appropriate but vague. The discussion could be strengthened by proposing specific "adjunctive protective approaches" (line 369), such as improved ventilation, prophylactic antivirals, or perhaps, next-generation mucosal vaccines.

Thank you. We have now added this information.

Reviewer #2: The authours have evaluated Older adult residents of long-term care facilities, and their infection/reinfection rates with Sara-CoV-2. While the maunscript is generally acceptable for publication and the data and conculsions are justified. I have some questions that may be considered.

Thank you for this assessment.

The manuscript puts a large amount of emphesis on the 4th to 5th vacine dose "Here we determined humoral and cellular immunity following delivery of a 5th vaccine dose" however Table 2 tells us that only 3 staff (of 168) and 64 residents (of 141) recived the 5th dose. in total only 22% of those in the study. Is it therefore correct to put such emphesis on the 4th to 5th vaccine dose when only 67 people of the 309 in the study actually recived this dose?. Also the inclusion of the Staff - who are a wholely different demographic - mostly much much younger than the residents - this also seems like an odd sub set to be included in the study - which doesnt really get an explanation.

Thank you. Very few staff received a 5th vaccine as they were generally much younger and were therefore not in the demographic group to receive additional booster vaccines. We have now discussed this limitation in the text.

The manuscript could also do with a quick grammar check.

Thank you. We have now reviewed the complete text and make it more accessible to readers.

Attachment Submitted filename: Response to Reviewers.docx

# Peer review: Decision Letter 1

**DOI:** 10.1371/journal.pone.0354079.r004

**Authors:** Mohammad Rashidul Hashan

6 May 2026

Dear Dr. Moss,

- Statistical interpretations raised by reviewer need careful consideration while revising the manuscript.

plosone@plos.org. When you're ready to submit your revision, log on to https://www.editorialmanager.com/pone/ and select the 'Submissions Needing Revision' folder to locate your manuscript file.

- A letter that responds to each point raised by the academic editor and reviewer(s). You should upload this letter as a separate file labeled 'Response to Reviewers'.
- A marked-up copy of your manuscript that highlights changes made to the original version. You should upload this as a separate file labeled 'Revised Manuscript with Track Changes'.
- An unmarked version of your revised paper without tracked changes. You should upload this as a separate file labeled 'Manuscript'.

If you would like to make changes to your financial disclosure, please include your updated statement in your cover letter. Guidelines for resubmitting your figure files are available below the reviewer comments at the end of this letter.

If applicable, we recommend that you deposit your laboratory protocols in protocols.io to enhance the reproducibility of your results. Protocols.io assigns your protocol its own identifier (DOI) so that it can be cited independently in the future. For instructions see: https://journals.plos.org/plosone/s/submission-guidelines#loc-laboratory-protocols. Additionally, PLOS ONE offers an option for publishing peer-reviewed Lab Protocol articles, which describe protocols hosted on protocols.io. Read more information on sharing protocols at https://plos.org/protocols?utm_medium=editorial-email&utm_source=authorletters&utm_campaign=protocols.

As the corresponding author, your ORCID iD is verified in the submission system and will appear in the published article. PLOS supports the use of ORCID, and we encourage all coauthors to register for an ORCID iD and use it as well. Please encourage your coauthors to verify their ORCID iD within the submission system before final acceptance, as unverified ORCID iDs will not appear in the published article. *Only*  the individual author can complete the verification step; PLOS staff *cannot*  verify ORCID iDs on behalf of authors.

We look forward to receiving your revised manuscript.

Kind regards,

Mohammad Rashidul Hashan, M.B.B.S.;Ph.D

Academic Editor

PLOS One

Journal Requirements:

If the reviewer comments include a recommendation to cite specific previously published works, please review and evaluate these publications to determine whether they are relevant and should be cited. There is no requirement to cite these works unless the editor has indicated otherwise.

[Note: HTML markup is below. Please do not edit.]

Reviewers' comments:

Reviewer's Responses to Questions

**Comments to the Author**

Reviewer #1: All comments have been addressed

Reviewer #3: (No Response)

**********

2. Is the manuscript technically sound, and do the data support the conclusions?

Reviewer #1: Yes

Reviewer #3: Yes

**********

3. Has the statistical analysis been performed appropriately and rigorously?

Reviewer #1: Yes

Reviewer #3: No

**********

4. Have the authors made all data underlying the findings in their manuscript fully available?

The PLOS Data policy

Reviewer #1: (No Response)

Reviewer #3: Yes

**********

5. Is the manuscript presented in an intelligible fashion and written in standard English?

Reviewer #1: Yes

Reviewer #3: Yes

**********

Reviewer #1: (No Response)

Reviewer #3: The aim of this study was to 1. Determine the antibody and cellular immune responses against SARS-CoV-2 following bivalent vaccination in long-term care facilities for older adults, and to 2. Assess breakthrough infection rates. The study uses a subset of VIVALDI cohort to compare immune responses after later booster doses (4th and 5th) to conclude that while the vaccines elicit robust systemic spike-specific immune responses, it does not prevent reinfection. The manuscript addresses an epidemiologically important question and has important public health implications for vulnerable older populations. However, the authors should consider these revisions to allow accurate interpretation of the data.

• It is unclear which participants were included in the analyses and in each figure. While the table nicely breaks down the population in the study, it doesn’t provide information about which samples actually ended up in the analyses and the breakdown of staff vs residents. For example, Figure 1A reports an n=41, are these all residents? Are these completely independent samples? Or are there repeated participants in each group? Similarly, Figures 4 and 6 have a different sample size compared to other figures with matched data. The manuscript can really benefit from mapping the included participants/samples into a CONSORT-style diagram and from noting when certain samples are omitted from analysis in specific figures. The sample sizes in matched data are very small. Why?

• Statistical analyses in all figures need reconsideration. In figure 1A and C, it is not clear if the analysis included repeated participants (i.e. same people contributed a sample after the 4th dose and after 5th dose). If participants are repeated, then the 4 groups are not fully independent so Kruskal–Wallis test is not appropriate, and a mixed-effects model should be used.

• In panels B and D, it would be nice to see a correlation graph between antibody titer after 4th and 5th dose instead of showing only matched data. Also, when were these matched samples collected for the 4th and 5th dose? Are equivalent time points being compared?

• 6–100 days is a very wide window. Immune responses at one week and at 3 months are not biologically equivalent, and you lose a lot of information. I suggest further stratifying the groups.

• Figure 2: Why were the Wuhan titers for prior infection samples analyzed with paired t test and the rest of the titers analyzed with Wilcoxon matched-pairs signed rank test? Were multiple comparisons done and corrected? Or were these unadjusted comparisons? The authors need to reanalyze and draw conclusions accordingly. From the current p values, bivalent vaccination broadened Omicron spike-binding responses more consistently in prior-infected than in infection-naïve participants. Also, similar to Figure 1, it should be stated whether the paired samples were taken at comparable times after each dose.

• Figure 3: again, statistical analysis. Were multiple comparisons done? What were the p values for each variant? How were the thresholds for weak and complete inhibition determined? Or are these the limits of detection of the assay? How did the authors reach the conclusion “bivalent vaccine enhances omicron variant-specific antibody titres irrespective of infection status and further enhances neutralisation capacity in donors who remain infection-naive”?

• Figure 4: The manuscript concludes that “Recognition of ancestral and BA.1 peptides was equivalent whilst those against BA.5 were slightly reduced, irrespective of prior infection status”. While cellular responses to BA.5 were modestly reduced in both prior-infected and infection-naive residents, the small sample size in infection-naive donors limits the precision of these estimates. Therefore, conclusions should be framed cautiously.

• Figure 5 is informative, but authors should be cautious about overstatements and implying causal dose thresholds and stabilization effects that are not directly tested by the statistical analysis presented. To evaluate changes in immune responses across doses and infection status groups, a mixed-effects longitudinal model would be more appropriate. Additionally, if this data is from all participants in the VIVALDI study, does that mean all ages are included?

• Figure 6: How many doses of vaccine did the participants in this figure have at that point? While Primary infection and reinfection events occurred, it is unclear how it was determined that the primary infection participants had “strong immunity”. Some of these participants were sitting at 10–100 BAU/mL in July, that is a low binding-antibody level. The data suggest that a subset of older donors remained weak humoral responders or had substantial waning in July, but with infection, immune responses were boosted. Overall, immunity in this cohort was shaped by both vaccination and repeated infection. In the resident reinfection group, infection did not result in further measurable boosting of spike antibody levels, likely reflecting a ceiling/ plateau effect or stable hybrid immunity.

Minor concerns:

• In addition to the absence of viral screening and inability to tell the time of prior infection with the use of anti-N IgG, it would strengthen the manuscript to acknowledge that anti-N IgGs decline more rapidly than spike antibodies, especially in older adults. Therefore, there could be some misclassification in the “naive group”.

• Additional clarification and description of the analytic structure and interpretation of results in the Results section would substantially improve readability and reduce ambiguity.

• Line 343, the text says August 2022 but the figure says July. Make sure it is consistent.

**********

what does this mean?). If published, this will include your full peer review and any attached files.

If you choose “no”, your identity will remain anonymous but your review may still be made public.

**Do you want your identity to be public for this peer review?**  For information about this choice, including consent withdrawal, please see our Privacy Policy

Reviewer #1: **Yes:** Oladayo A. Oyebanji

Reviewer #3: No

**********

[NOTE: If reviewer comments were submitted as an attachment file, they will be attached to this email and accessible via the submission site. Please log into your account, locate the manuscript record, and check for the action link "View Attachments". If this link does not appear, there are no attachment files.]

To ensure your figures meet our technical requirements, please review our figure guidelines: https://journals.plos.org/plosone/s/figures

You may also use PLOS’s free figure tool, NAAS, to help you prepare publication quality figures: https://journals.plos.org/plosone/s/figures#loc-tools-for-figure-preparation.

NAAS will assess whether your figures meet our technical requirements by comparing each figure against our figure specifications.

# Author comment: Author response to Decision Letter 2

**DOI:** 10.1371/journal.pone.0354079.r005

27 Jun 2026

PONE-D-25-51786

Second response to Reviewers

Comments to the Author

1. If the authors have adequately addressed your comments raised in a previous round of review and you feel that this manuscript is now acceptable for publication, you may indicate that here to bypass the “Comments to the Author” section, enter your conflict of interest statement in the “Confidential to Editor” section, and submit your "Accept" recommendation.

Reviewer #1: All comments have been addressed

Reviewer #3: (No Response)

2. Is the manuscript technically sound, and do the data support the conclusions?

The manuscript must describe a technically sound piece of scientific research with data that supports the conclusions. Experiments must have been conducted rigorously, with appropriate controls, replication, and sample sizes. The conclusions must be drawn appropriately based on the data presented.

Reviewer #1: Yes

Reviewer #3: Yes

3. Has the statistical analysis been performed appropriately and rigorously?

Reviewer #1: Yes

Reviewer #3: No

4. Have the authors made all data underlying the findings in their manuscript fully available?

The PLOS Data policy requires authors to make all data underlying the findings described in their manuscript fully available without restriction, with rare exception (please refer to the Data Availability Statement in the manuscript PDF file). The data should be provided as part of the manuscript or its supporting information, or deposited to a public repository. For example, in addition to summary statistics, the data points behind means, medians and variance measures should be available. If there are restrictions on publicly sharing data—e.g. participant privacy or use of data from a third party—those must be specified.

Reviewer #1: (No Response)

Reviewer #3: Yes

5. Is the manuscript presented in an intelligible fashion and written in standard English?

PLOS ONE does not copyedit accepted manuscripts, so the language in submitted articles must be clear, correct, and unambiguous. Any typographical or grammatical errors should be corrected at revision, so please note any specific errors here.

Reviewer #1: Yes

Reviewer #3: Yes

6. Review Comments to the Author

Please use the space provided to explain your answers to the questions above. You may also include additional comments for the author, including concerns about dual publication, research ethics, or publication ethics. (Please upload your review as an attachment if it exceeds 20,000 characters)

Reviewer #1: (No Response)

Reviewer #3: The aim of this study was to 1. Determine the antibody and cellular immune responses against SARS-CoV-2 following bivalent vaccination in long-term care facilities for older adults, and to 2. Assess breakthrough infection rates. The study uses a subset of VIVALDI cohort to compare immune responses after later booster doses (4th and 5th) to conclude that while the vaccines elicit robust systemic spike-specific immune responses, it does not prevent reinfection. The manuscript addresses an epidemiologically important question and has important public health implications for vulnerable older populations. However, the authors should consider these revisions to allow accurate interpretation of the data.

Thank you for advising on these potential revisions. We have undertaken these and have made substantial statistical revisions throughout the paper. These have further improved the manuscript and we thank the reviewer for the advice.

• It is unclear which participants were included in the analyses and in each figure. While the table nicely breaks down the population in the study, it doesn’t provide information about which samples actually ended up in the analyses and the breakdown of staff vs residents. For example, Figure 1A reports an n=41, are these all residents? Are these completely independent samples? Or are there repeated participants in each group? Similarly, Figures 4 and 6 have a different sample size compared to other figures with matched data. The manuscript can really benefit from mapping the included participants/samples into a CONSORT-style diagram and from noting when certain samples are omitted from analysis in specific figures. The sample sizes in matched data are very small. Why?

Thank you for this. The study was undertaken using small sample collections from frail residents in care homes and sometimes not enough cells or plasma was available for study. Laboratory analysis was also undertaken on two sites to maximise expertise. However, we have now made the limitation on sample size and pairing the major focus in our section on the limitations of the study in the Discussion section.

• Statistical analyses in all figures need reconsideration. In figure 1A and C, it is not clear if the analysis included repeated participants (i.e. same people contributed a sample after the 4th dose and after 5th dose). If participants are repeated, then the 4 groups are not fully independent so Kruskal–Wallis test is not appropriate, and a mixed-effects model should be used.

Thank you for this valuable advice.

A mixed effects model has been applied to Figures 1A and 1C. A new figure has been replaced in this regard. The figure legends have been amended appropriately. This analysis removes a prior statistical difference between the groups. However, the PI model showed that the Spike over time was significantly different with a statistical significance in early 4th and later 5th and early 5th and late 5th groups.

• In panels B and D, it would be nice to see a correlation graph between antibody titer after 4th and 5th dose instead of showing only matched data. Also, when were these matched samples collected for the 4th and 5th dose? Are equivalent time points being compared?

• 6–100 days is a very wide window. Immune responses at one week and at 3 months are not biologically equivalent, and you lose a lot of information. I suggest further stratifying the groups.

Thank you for this advice. It is indeed a wide window and this has been added to the limitations of the manuscript.

• Figure 2: Why were the Wuhan titers for prior infection samples analyzed with paired t test and the rest of the titers analyzed with Wilcoxon matched-pairs signed rank test? Were multiple comparisons done and corrected? Or were these unadjusted comparisons? The authors need to reanalyze and draw conclusions accordingly. From the current p values, bivalent vaccination broadened Omicron spike-binding responses more consistently in prior-infected than in infection-naïve participants. Also, similar to Figure 1, it should be stated whether the paired samples were taken at comparable times after each dose.

Thank you for this. Wilcoxon matched-pairs has been performed on the figure.

Comparisons were restricted to five pre-specified SARS-CoV-2 variants selected a priori for their biological relevance. These analyses were hypothesis-driven rather than exploratory, with each comparison addressing a predefined research question rather than representing a broad, data-driven search for associations. Consequently, we did not apply a multiple-testing correction, as our understanding is that such adjustments are generally intended to control the false-positive rate in exploratory analyses involving large numbers of simultaneous comparisons and may be unnecessarily conservative for such a small set of pre-specified hypotheses.

• Figure 3: again, statistical analysis. Were multiple comparisons done? What were the p values for each variant? How were the thresholds for weak and complete inhibition determined? Or are these the limits of detection of the assay? How did the authors reach the conclusion “bivalent vaccine enhances omicron variant-specific antibody titres irrespective of infection status and further enhances neutralisation capacity in donors who remain infection-naive”?

Thank you.

Thresholds for weak and complete inhibition were determined by manufacturers’ guidance. Comparisons were restricted to five pre-specified SARS-CoV-2 variants selected a priori for their biological relevance. These analyses were hypothesis-driven rather than exploratory, with each comparison addressing a predefined research question rather than representing a broad, data-driven search for associations.

Consequently, we did not apply a multiple-testing correction, as such adjustments are generally intended to control the false-positive rate in exploratory analyses involving large numbers of simultaneous comparisons and may be unnecessarily conservative for such a small set of pre-specified hypotheses.

Wilcoxon matched-pairs has now been performed on the figure

• Figure 4: The manuscript concludes that “Recognition of ancestral and BA.1 peptides was equivalent whilst those against BA.5 were slightly reduced, irrespective of prior infection status”. While cellular responses to BA.5 were modestly reduced in both prior-infected and infection-naive residents, the small sample size in infection-naive donors limits the precision of these estimates. Therefore, conclusions should be framed cautiously.

Thank you for spotting this. We agree that we over interpreted this effect. This has been rewritten accordingly.

• Figure 5 is informative, but authors should be cautious about overstatements and implying causal dose thresholds and stabilization effects that are not directly tested by the statistical analysis presented. To evaluate changes in immune responses across doses and infection status groups, a mixed-effects longitudinal model would be more appropriate. Additionally, if this data is from all participants in the VIVALDI study, does that mean all ages are included?

Thank you. We have now amended the text to say that. “However, this representation is for information only and, as the data have not been tested by statistical analysis, causal dose thresholds and stabilization effects cannot be interpreted”.

• Figure 6: How many doses of vaccine did the participants in this figure have at that point? While Primary infection and reinfection events occurred, it is unclear how it was determined that the primary infection participants had “strong immunity”. Some of these participants were sitting at 10–100 BAU/mL in July, that is a low binding-antibody level. The data suggest that a subset of older donors remained weak humoral responders or had substantial waning in July, but with infection, immune responses were boosted. Overall, immunity in this cohort was shaped by both vaccination and repeated infection. In the resident reinfection group, infection did not result in further measurable boosting of spike antibody levels, likely reflecting a ceiling/ plateau effect or stable hybrid immunity.

Thank you. Recipients had received at least 3 vaccines. We have rewritten this section based on the reviewers comments.

Minor concerns:

• In addition to the absence of viral screening and inability to tell the time of prior infection with the use of anti-N IgG, it would strengthen the manuscript to acknowledge that anti-N IgGs decline more rapidly than spike antibodies, especially in older adults. Therefore, there could be some misclassification in the “naive group”.

Thank you. This is an important point and this has been more clearly stated in the limitations section.

• Additional clarification and description of the analytic structure and interpretation of results in the Results section would substantially improve readability and reduce ambiguity.

Thank you. This has been added to the Methods section.

• Line 343, the text says August 2022 but the figure says July. Make sure it is consistent.

We apologise for this error which has been corrected.

Attachment Submitted filename: Response_to_Reviewers_auresp_2.docx

# Editor report: Decision Letter 2

**DOI:** 10.1371/journal.pone.0354079.r006

**Authors:** Mohammad Rashidul Hashan

2 Jul 2026

High rates of SARS-CoV-2 reinfection in residents of long term care facilities despite robust spike-specific immunity following serial vaccination

PONE-D-25-51786R2

Dear Dr. Paul,

We’re pleased to inform you that your manuscript has been judged scientifically suitable for publication and will be formally accepted for publication once it meets all outstanding technical requirements.

Within one week, you’ll receive an e-mail detailing the required amendments. When these have been addressed, you’ll receive a formal acceptance letter and your manuscript will be scheduled for publication.

An invoice will be generated when your article is formally accepted. Please note, if your institution has a publishing partnership with PLOS and your article meets the relevant criteria, all or part of your publication costs will be covered. Please make sure your user information is up-to-date by logging into Editorial Manager at Editorial Manager® and clicking the ‘Update My Information' link at the top of the page. For questions related to billing, please contact billing support.

If your institution or institutions have a press office, please notify them about your upcoming paper to help maximize its impact. If they’ll be preparing press materials, please inform our press team as soon as possible -- no later than 48 hours after receiving the formal acceptance. Your manuscript will remain under strict press embargo until 2 pm Eastern Time on the date of publication. For more information, please contact onepress@plos.org.

Kind regards,

Mohammad Rashidul Hashan, M.B.B.S.;Ph.D

Academic Editor

PLOS One

Additional Editor Comments (optional):

Reviewers' comments:

# Editor report: Acceptance letter

**DOI:** 10.1371/journal.pone.0354079.r007

**Authors:** Mohammad Rashidul Hashan

PONE-D-25-51786R2

PLOS One

Dear Dr. Moss,

I'm pleased to inform you that your manuscript has been deemed suitable for publication in PLOS One. Congratulations! Your manuscript is now being handed over to our production team.

At this stage, our production department will prepare your paper for publication. This includes ensuring the following:

* All references, tables, and figures are properly cited

* All relevant supporting information is included in the manuscript submission,

* There are no issues that prevent the paper from being properly typeset

You will receive further instructions from the production team, including instructions on how to review your proof when it is ready. Please keep in mind that we are working through a large volume of accepted articles, so please give us a few days to review your paper and let you know the next and final steps.

Lastly, if your institution or institutions have a press office, please let them know about your upcoming paper now to help maximize its impact. If they'll be preparing press materials, please inform our press team within the next 48 hours. Your manuscript will remain under strict press embargo until 2 pm Eastern Time on the date of publication. For more information, please contact onepress@plos.org.

You will receive an invoice from PLOS for your publication fee after your manuscript has reached the completed accept phase. If you receive an email requesting payment before acceptance or for any other service, this may be a phishing scheme. Learn how to identify phishing emails and protect your accounts at https://explore.plos.org/phishing.

If we can help with anything else, please email us at customercare@plos.org.

Thank you for submitting your work to PLOS ONE and supporting open access.

Kind regards,

PLOS ONE Editorial Office Staff

on behalf of

Dr. Mohammad Rashidul Hashan

Academic Editor

PLOS One
